# EHMTI-0068. Facial plastic surgery for migraine therapy: personal procedures

**DOI:** 10.1186/1129-2377-15-S1-G6

**Published:** 2014-09-18

**Authors:** G Caruana, N Bertozzi, MP Grieco, E Raposio

**Affiliations:** 1 Surgical Science, University of Parma, Parma, Italy

## Background

During the last few years multiple studies have demonstrated the efficacy of surgical treatment in patients who suffer from migraine headache: by removing the hyperactive surrounding muscles, the trigger point is being eliminated.

## Aim

The aim of this study was to demonstrate the efficacy of surgical decompression by means of both endoscopic and open surgery, through an innovative and improved technique compared with beforehand evaluated surgical techniques.

## Method

Fifty-one patients who complained of chronic migraine headaches underwent a frontal bilateral selective miotomy procedure of Procerus, Depressor Supercilii and Corrugator Supercilii Muscles by means of video-assisted endoscopic surgery, and/or an occipital selective miotomy procedure of Occipital, Trapezius, Sternocleidomastoid and Semispinalis Capitis Muscles by means of open surgery.

## Results

Of the 51 patients included in the study (range, 18 to 73 years), 38 were women and 13 were men. Forty-four of 51 patients (86.3%) reported a positive response to the surgery: 21 of 51 patients (41.2%) observed complete elimination, 23 patients (45.1%) experienced significant improvement (at least 50% reduction in intensity or frequency), and 7 patients (13.7%) did not notice a change in their migraine headaches.

## Conclusion

This study confirms previous data in literature, strengthening the role of a peripheral mechanism (trigger points) in migraine headaches. Moreover, the minimally invasive procedure we described, is easy, fast and cost-effective, relying on the use of a single instrument, also reducing the numbers of postoperative scars from five to one.

No conflict of interest.

